# Conversion of T2-Weighted Magnetic Resonance Images of Cervical Spine Trauma to Short T1 Inversion Recovery (STIR) Images by Generative Adversarial Network

**DOI:** 10.7759/cureus.60381

**Published:** 2024-05-15

**Authors:** Atsushi Yunde, Satoshi Maki, Takeo Furuya, Sho Okimatsu, Takaki Inoue, Masataka Miura, Yuki Shiratani, Yuki Nagashima, Juntaro Maruyama, Yasuhiro Shiga, Kazuhide Inage, Yawara Eguchi, Sumihisa Orita, Seiji Ohtori

**Affiliations:** 1 Department of Orthopaedic Surgery, Chiba University, Graduate School of Medicine, Chiba, JPN

**Keywords:** t2-weighted magnetic resonance images, short t1 inversion recovery, image conversion, generated adversarial network, deep learning, cervical spine trauma, magnetic resonance imaging

## Abstract

Introduction: The short T1 inversion recovery (STIR) sequence is advantageous for visualizing ligamentous injuries, but the STIR sequence may be missing in some cases. The purpose of this study was to generate synthetic STIR images from MRI T2-weighted images (T2WI) of patients with cervical spine trauma using a generative adversarial network (GAN).

Methods: A total of 969 pairs of T2WI and STIR images were extracted from 79 patients with cervical spine trauma. The synthetic model was trained 100 times, and the performance of the model was evaluated with five-fold cross-validation.

Results: As for quantitative validation, the structural similarity score was 0.519±0.1 and the peak signal-to-noise ratio score was 19.37±1.9 dB. As for qualitative validation, the incorporation of synthetic STIR images generated by a GAN alongside T2WI substantially enhances sensitivity in the detection of interspinous ligament injuries, outperforming assessments reliant solely on T2WI.

Conclusion: The GAN model can generate synthetic STIRs from T2 images of cervical spine trauma using image-to-image conversion techniques. The use of a combination of synthetic STIR images generated by a GAN and T2WI improves sensitivity in detecting interspinous ligament injuries compared to assessments that use only T2WI.

## Introduction

Magnetic resonance imaging (MRI) is important for the initial assessment of a traumatic spinal cord injury (SCI) [1]. The role of MRI in the evaluation of the acutely injured spine is well established and contributes to the assessment of SCI as well as vertebral body and ligamentous injuries [2]. A short T1 inversion recovery (STIR) sequence is most useful for visualizing ligamentous injuries [3,4]. However, a STIR sequence may not always be acquired due to restrictions on scan time or a lack of a physician’s order for the sequence. Long MRI durations to obtain multiple different sequences can put the patient's life at risk because patients with cervical SCI or polytrauma have unstable respiratory and hemodynamic conditions. Reducing MRI scan time is important for the imaging evaluation of patients with cervical spine trauma.

In recent years, remarkable developments in deep learning technologies have been made and put to practical use in various fields, including medical imaging. While it is common to use deep learning techniques to classify, detect, or segment an existing image, neural networks that can generate new images have attracted attention [5]. In recent years, the use of conditional generative adversarial networks (GANs) has become the standard for supervised image-to-image conversion [6]. Generators and discriminators are the two networks that make up a GAN. This method involves training both the generator and discriminator, which then compete against one another. The generator tries to generate a new image that resembles the target image. The discriminator tries to distinguish between the actual and artificially generated images. Finally, the generated images should be essentially indistinguishable from the actual images extracted from the true dataset. Pix2pix, a type of GAN, is an image transformation algorithm technique that learns the relationship between the image pairs and uses a single image to create a new image pair [7]. 

Several studies have been done using deep learning to reduce MRI scan times. A conditional GAN-based deep learning method for fast MRI reconstruction has been proposed to significantly reduce the processing time from seconds to milliseconds per 2D slice [8]. A new method involving a multi-level, densely connected super-resolution network with GAN-guided training has been reported to reduce scan times by a factor of four while maintaining approximately the same image resolution and quality [9].

In this study, we aimed to generate synthetic STIR images from MR T2-weighted images (T2WI) of patients with cervical spine trauma using a GAN. We evaluated the quality of the synthetic STIR images in both quantitative and qualitative ways.

## Materials and methods

Patients

This study was approved by the Institutional Review Board of the Chiba University Graduate School of Medicine, Chiba, Japan, and the requirement for informed consent was waived due to the retrospective analysis (approval number: 3329). All procedures involving human participants were in accordance with the 1964 Declaration of Helsinki and its later amendments. This retrospective study included 290 patients with cervical spine trauma seen at our hospital from January 2015 to May 2021. Patients with cervical spine trauma who underwent MRI, including both sagittal T2WI sequences and STIR sagittal sequences of the cervical spine after trauma, were eligible for inclusion in this study. A total of 212 out of 290 patients were excluded because either the T2WI or STIR sequence was missing, resulting in 79 patients. 

Dataset

Our dataset mainly used MRI images taken under consistent conditions, with examinations performed on 1.5 Tesla (T) or 3.0 T MR systems. Although the T2WI and STIR of the MRI acquisition protocol were not standardized, the following parameters were generally used: T2WI; repetition time/echo time = 3000-5000/100-110 ms; flip angle = 180°; slice thickness = 3.0 mm; field-of-view = 240 × 240 mm2; matrix size = 224-288 × 224-288, STIR; repetition time/echo time = 3000-5000/40-70 ms; flip angle =90°; slice thickness = 3.0 mm; field-of-view = 240 × 240 mm2; matrix size = 224-320 × 224-320.

Our study used five MRI scanners from different vendors, including 1.5 and 3.0T models, within our hospital. Our dataset not only included the diversity found within our own facility but also incorporated data from patients referred to us who had their MRI scans performed at other institutions. Although collecting data from a single scanner would have been ideal for generating cleaner images using GAN, we prioritized having a larger dataset over scanner uniformity. This approach was essential to ensuring our research was based on a sufficient amount of data and accurately represented the varied clinical environments encountered in practice. Digital Imaging and Communications in Medicine (DICOM) MR images of cervical spine trauma were converted to jpeg images after standardizing the window level and width for the corresponding images on the T2WI and STIR. In the training dataset, all identical slices of T2WI and STIR images obtained from each patient were paired [7]. The training dataset included 969 pairs of images from 79 patients. To maximize the size of the training dataset, a total of 10 to 15 images were used for each patient, including not only the midsagittal image of the cervical spine but also the adjacent lateral slices. We carefully chose lateral slices up to where the vertebral pedicles are fully visible, creating a focused and broad dataset for training our GAN model. This choice balanced showing body structures accurately with the practical needs of model training. To evaluate the performance of the GAN in creating STIR images, we employed a five-fold cross-validation method. The MR images of cervical spine trauma were randomly divided into five independent subgroups of equal size. The images from the four subgroups were used as the training dataset, and the remaining independent subgroup was used as the validation dataset.

Generating synthesis

The GAN model was trained and validated using a computer with a GeForce RTX 3090 graphics processing unit (NVIDIA, Santa Clara, CA), a Core i9-10900KF 3.7 GHz central processing unit (Intel, Santa Clara, CA), and 32 GB of random-access memory. The GAN model architecture was constructed using Python, version 3.6.7 (https://www.python.org), and Keras, version 2.4.3 with Tensorflow 2.1.0. The image synthesis was constructed with Pix2pix for image-to-image translation. Pix2pix was the first general-purpose GAN-based image-to-image conversion framework developed by Isola et al. [10]. The input image data were set to 512 × 256 pixels, and the output image data were set to 700 × 700 pixels. After building the models, we generated images with 100 epochs. 

Evaluations of synthetic images

Of the 79 cases of cervical spine trauma, 79 midsagittal images were used for the evaluation. However, in the training dataset, not only the midsagittal image of the cervical spine but also the adjacent lateral slices were used.

**Figure 1:**
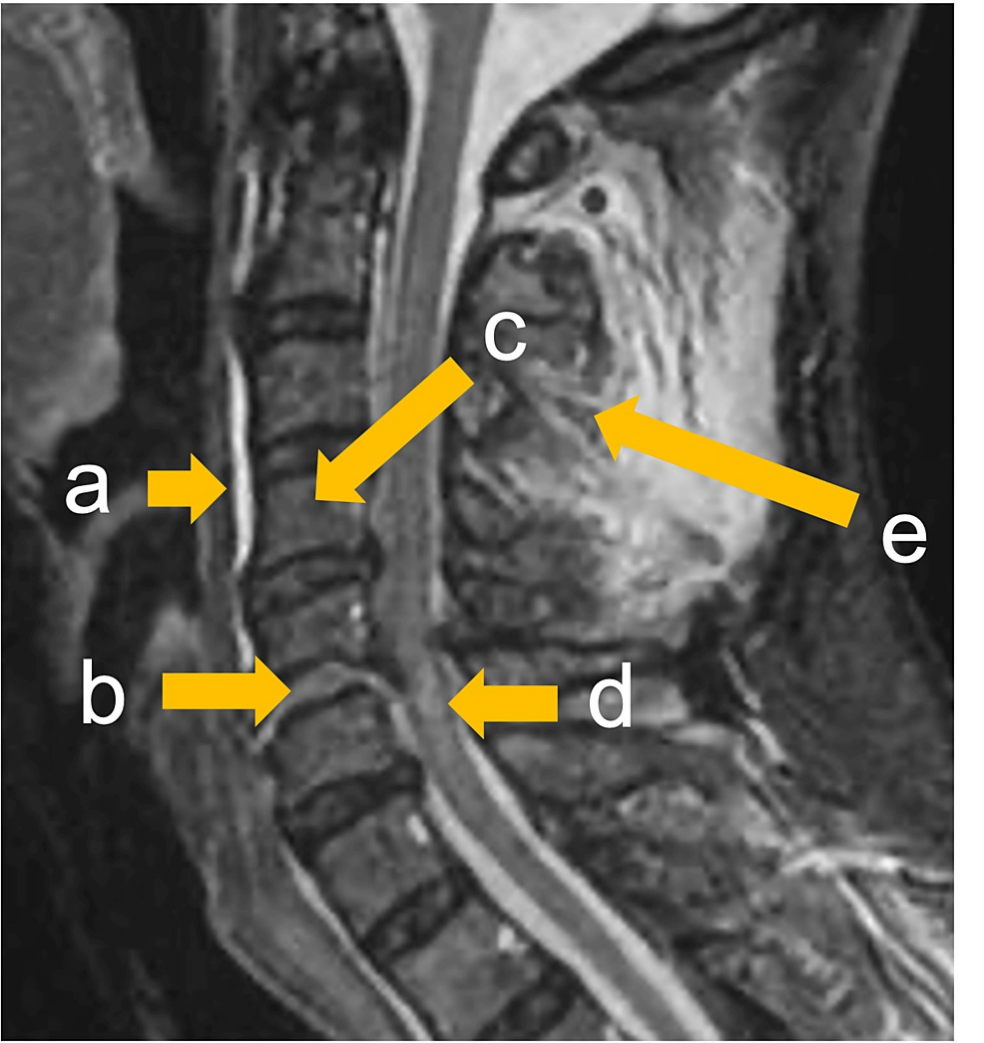
Anatomical position on the MR sagittal image of a patient with cervical spine trauma The letters indicate the anatomical position on the MR sagittal image of a patient with cervical spine trauma: prevertebral soft tissue (a), intervertebral disc (b), vertebral column (c), spinal cord (d), and interspinous ligament (e).

The evaluation was both quantitative and qualitative. For the quantitative validation, we used two indicators to verify the generated images. The peak signal-to-noise ratio (PSNR) is widely used for the quantitative evaluation of medical images. Higher values indicate better image quality and are generally around 30-50 dB. The second indicator was the structural similarity index (SSIM), which is used for visual image quality assessment and evaluates the overall structure of the image. The value of this index ranges from 0 to one, and the higher the value, the closer the image structure is to that of the actual image [11]. For the qualitative validation, the presence or absence of the injury in the prevertebral soft tissue, intervertebral disc, vertebral column, spinal cord, and interspinous ligament was determined based on the signal intensity change on the T2WI and the synthetic and actual STIR MRIs of the cervical spine trauma. An actual STIR MR image showing the anatomy of the cervical spine is shown in Figure 1. 

Initially, three spine surgeons, two of them with six years of experience each and another with eight years of experience, evaluated T2WI to identify any changes in signal intensity for various spinal structures. Next, both synthetic STIR images and T2WI images were used in a combined assessment to detect any tissue damage. Finally, the findings were compared in two sets: one comparing T2WI to actual STIR images, and another comparing the combination of T2WI and synthetic STIR images to actual STIR images. The sensitivity of the results was then calculated for each set.

Statistical analysis 

JMP software (version 15.0.0; SAS Institute Inc., Cary, NC) was used to conduct all statistical analyses. A McNemar test was used to compare the sensitivity between T2WI and T2WI with synthetic STIR images. The information was presented in the form of a mean and a standard deviation. Statistical significance was defined as a p-value of less than 0.05.

## Results

Prevalence of the injured anatomical part of the cervical spine 

The prevalence of the injured anatomical part of the cervical spine is shown in Table 1. One spine surgeon with 10 years of experience determined the presence of the intensity change in the anatomical part of the cervical spine on the STIR MR image, which served as the ground truth. 

**Table 1:** Prevalence of the injured anatomical part of the cervical spine The letters indicate the anatomical position on the MR sagittal image of a patient with cervical spine trauma: prevertebral soft tissue (a), intervertebral disc (b), vertebral column (c), spinal cord (d), and interspinous ligament (e).

Anatomical part of the cervical spine	Ground truth (%)
Prevertebral soft tissue	39.0
Intervertebral disc	3.8
Vertebral column	2.8
Spinal cord	10.3
Interspinous ligament	35.4

Quantitative evaluation

The quantitative validation of the synthetic STIR images indicated a PSNR score of 19.37±1.9 dB and an SSIM score of 0.519±0.1.

Qualitative evaluation

The qualitative results are indicated in Table 2. There were no significant differences in the concordance rate of the MRI intensity between the T2WI and T2WI with synthetic STIR images in the prevertebral soft tissue, intervertebral disc, vertebral body, or spinal cord. In contrast, the sensitivity in detecting interspinous injuries significantly increased when using a combination of synthetically generated STIR images by a GAN and T2WI, compared to assessments relying solely on T2WI, among all spine surgeons (P <0.0001). 

**Table 2:** Comparison of diagnostic sensitivity in detecting interspinous ligament injuries between T2WI-only and combined T2WI and synthetic STIR approaches. A McNemar test was used to compare the sensitivity in detecting interspinous ligament injuries between T2WI-only and combined T2WI and synthetic STIR. ✻P <.05 MRI: magnetic resonance imaging; T2WI: T2-weighted images

Evaluator	Modality of MRI	Sensitivity (%)				
		Prevertebral soft tissue	Intervertebral disc	Vertebral column	Spinal cord	Interspinous ligamentous
Spine surgeon 1	T2WI-only	74.3	73.3	45.5	95	48.6
	T2WI and synthetic STIR	76.3	86.7	54.5	100	81.9
	P-value	0.69	0.36	0.67	0.15	<0.0001*
Spine surgeon 2	T2WI-only	44.1	52.9	9.1	72.5	25.3
	T2WI and synthetic STIR	49.3	58.8	9.1	80	81.9
	P-value	0.36	0.73	1	0.43	<0.0001*
Spine surgeon 3	T2WI-only	22.4	13.3	18.2	70	3.6
	T2WI and synthetic STIR	23.7	33.3	27.3	72.5	52.2
	P-value	0.79	0.2	0.61	0.8	<0.0001*

**Figure 2:**
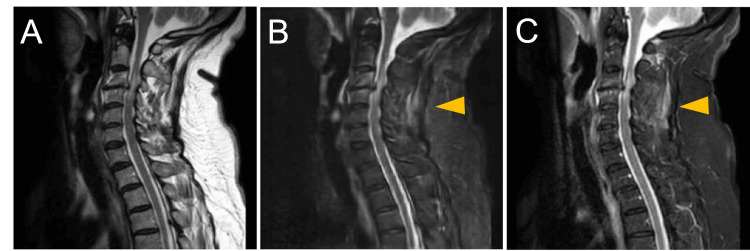
A representative case featuring images generated by a GAN (A) A T2-weighted image of a spinal cord injury: the interspinous ligament injury is not obvious; (B) A synthetic STIR image of SCI: the high-intensity changes are seen in the interspinous ligament (yellow arrowhead); (C) An actual STIR image of SCI: the high-intensity changes are seen in the interspinous ligament (yellow arrowhead). SCI: spinal cord injury; STIR: short T1 inversion recovery

Representative images are shown in Figure 2. The actual MRI STIR image shows interspinous ligament damage, but the MRI T2WI does not show obvious interspinous ligament damage. Synthetic STIR images, on the other hand, reproduced the interspinous ligament injury.

Reliability and accuracy 

The inter-rater intraclass correlation coefficient (ICC) for evaluations based on T2WI alone and in combination with synthetic STIR images underscored the moderate agreement among clinicians, with ICCs of 0.31 and 0.44, respectively. The variability in false positive rates (19.8% and 52.0% for the first examiner, 10.7% and 37.3% for the second examiner, and 1.6% and 22.6% for the third examiner) when evaluating T2WI alone versus in combination with synthetic STIR highlights aspects for further model improvement and emphasizes the importance of continued collaboration with clinical experts to enhance accuracy.

## Discussion

In the present study, our model successfully generated synthetic STIR images from T2WI of patients with cervical spine trauma using a GAN. A qualitative evaluation indicated that using a combination of synthetic STIR images generated by a GAN and T2WI improves sensitivity in detecting interspinous ligament injuries compared to assessments that use only T2WI. The quantitative evaluation of the synthetic STIR images was fair.

This is the first study in which the cross-modality synthesis of STIR images from T2WI in patients with cervical spine trauma using a GAN was successfully achieved. So far, research on anatomically normal image generation has been the main focus, and there are few studies of image generation in patients with injuries or diseases. Previous image synthesis studies in the spine using Pix2Pix resulted in conditional GAN standard image-to-image modality conversions of the lumbar spine, such as T1-weighted images (T1WI) to T2WI and T2WI to STIR images, or the automatic conversion between different image modalities, such as sagittal X-ray to T2WI [12]. An image synthesis study of the knee demonstrated the generation of STIR images from three alternative MR image sequences (T1W1, T2WI, and gradient-recalled echo) using a deep neural network. Their method scored better in a subjective assessment by a musculoskeletal radiologist compared to other image generation methods such as Replica [13], Multimodal Imaging [14], and Pix2pix [15]. A similar deep learning model of spine images with 15 different categories of pathology was recently published using T1WI and T2WI MR to generate STIR sequences. They showed good similarity between the real STIR and the generated STIR images, including the various pathologies, although they did not conduct a detailed validation for each disease [16]. In the present study, instead of aiming for general image-to-image conversion in healthy controls, we used a GAN to customize the method for a specific application. With the use of images from patients with cervical spine trauma instead of normal images as the training data for the CNN, our GAN model was able to generate images of cervical spine trauma. 

In this study, the GAN model increased the sensitivity of interspinous ligament injury detection. This may be due to the large number of images showing interspinous ligament injuries that were used in the training dataset. The detection of interspinous ligament injury is important because the development of spinal cord injury is correlated with the grade of soft tissue injury [17]. In addition, a posterior cervical ligament injury is one of the elements determining the subaxial cervical spine injury classification for surgical indication [18].

In an investigation involving the transformation of CT images into MRI images through the utilization of cycle GAN, PSNR and SSIM values at Epoch 100 were reported as 17.761±2.497dB and 0.585±0.040, respectively. In the current study, the corresponding PSNR and SSIM values were 19.37±1.9dB and 0.519±0.1, indicating a reasonably satisfactory outcome [19]. Nevertheless, alternate investigations have documented superior values compared to those observed in this study. Although the interspinous ligament injury was well reproduced in the synthetic STIR image, an inaccurate reproduction of the intensity changes in the intervertebral disc and posterior longitudinal ligament (PLL) was considered to be the cause of the relatively low values for the PSNR and SSIM. As many as 35% of patients had interspinal ligament injuries, which was the reason for the excellent reproduction of interspinous ligament intensity changes. However, only 4%-5% of patients had disc injuries, making it difficult to reproduce the intensity changes due to disc and ligament injuries on the synthetic STIR images. As a result, the PSNR and SSIM scores were low.

One major advantage of applying a GAN to MR image analysis is the possibility of reducing the time required to acquire multiple sequences. A GAN has the potential to reduce MR acquisition time by generating the target sequence from the sequences already acquired. For pathology such as cervical spine trauma where MRI examination time is limited [20], the use of GAN-generated images would be expected to reduce MR scan time and re-scan rate. 

This research has the potential to advance clinical practice by improving the efficiency and accuracy of MRI diagnostics for cervical spine injuries, providing a faster, less invasive diagnostic tool that may considerably improve patient outcomes and optimize healthcare workflows.

The present study has several limitations. First, the number of patients and images included in the present study was small. It is necessary to further increase the number of images to train the model to improve the synthesized STIR images. Second, the prevalence of the anatomical site of the cervical spine injury was imbalanced. There were few patients with injuries to the vertebral bodies and intervertebral discs, and thus, injuries to these anatomical sites were difficult to reproduce in the generated images. Third, we only used midline images. These images are important for diagnosing spine problems, but focusing on them might not show everything. We plan to examine more types of images in the future to improve our GAN model for better clinical utility and to ensure its robustness in various scenarios.

## Conclusions

The findings of this study highlight the potential of GANs to transform T2WI of cervical spine trauma into STIR images. The capability of the GAN model to synthesize STIR images that facilitate enhanced detection of interspinous ligament injuries marks a significant step forward in medical imaging. This advancement is particularly valuable in clinical settings where MRI scan time is critical and must be minimized due to patient conditions. The application of GANs not only helps in reducing the total MRI scan duration but also ensures that high-quality diagnostic images are available even when certain sequences cannot be obtained directly due to time constraints or other factors.

The use of synthetic STIR images alongside traditional T2WI has been shown to improve sensitivity in detecting critical ligament injuries, which are often difficult to discern with T2WI alone. This could potentially lead to better diagnostic accuracy and more informed decision-making in the treatment of cervical spine injuries. Additionally, extending this technology to other types of medical imaging could revolutionize diagnostics across various domains of medicine, enhancing both the efficiency and effectiveness of medical interventions.
